# Prostate-specific loss of UXT promotes cancer progression

**DOI:** 10.18632/oncotarget.26573

**Published:** 2019-01-22

**Authors:** Yu Wang, Eric D. Schafler, Phillip A. Thomas, Susan Ha, Gregory David, Emily Adney, Michael J. Garabedian, Peng Lee, Susan K. Logan

**Affiliations:** ^1^ Department of Urology, New York University School of Medicine, New York 10016, NY, USA; ^2^ Department of Microbiology, New York University School of Medicine, New York 10016, NY, USA; ^3^ Department of Biochemistry and Molecular Pharmacology, New York University School of Medicine, New York 10016, NY, USA; ^4^ Institute for Systems Genetics, New York University School of Medicine, New York 10016, NY, USA; ^5^ Department of Pathology, New York Harbor Healthcare System, New York 10010, NY, USA; ^6^ McKusick-Nathans Institute of Genetic Medicine, Johns Hopkins University School of Medicine, Baltimore 21205, MD, USA

**Keywords:** UXT, retroelement, prostate cancer

## Abstract

Ubiquitously-expressed, prefoldin-like chaperone (UXT) also called Androgen Receptor Trapped clone-27 (ART-27) is widely expressed in human tissues. Our previous studies showed that UXT regulates transcription repression including androgen receptor (AR) signaling in prostate cancer. Here we analyzed a tissue microarray consisting of normal prostate, benign prostatic hyperplasia, high grade prostatic intraepithelial neoplasia (HGPIN) and primary prostate cancer cases for UXT protein expression. We found that HGPIN and malignant tumors have significantly decreased UXT expression compared to the normal prostate. Loss of UXT expression in primary prostate cancer is positively associated with high Gleason grade and poor relapse-free survival. We engineered prostate-specific *Uxt*^*KO*^ mice that developed a hyperplastic phenotype with apparent prostate secretion fluid blockage as well as PIN by 4-6 months. Doubly mutant *Uxt*^*KO*^/*Pten*^*KO*^ mice developed a more aggressive PIN phenotype. UXT depletion in prostate cancer cells also increased retroelements expression, including LINE-1 and Alu. Consistent with this finding *Uxt*^*KO*^ mice have increased LINE-1 protein levels in the prostate compared to control mice. In addition, cancer cells with UXT depletion have increased retrotransposition activity and accumulated DNA damage. Our findings demonstrate that loss of UXT is an early event during prostate cancer progression, which may contribute to genome instability.

## INTRODUCTION

The androgen receptor (AR) pathway plays a vital role in prostate cancer (CaP) growth. Therefore, blocking this pathway through androgen deprivation and AR antagonists is the mainstay of prostate cancer treatment. However, prostate cancer patients receiving androgen deprivation therapy often relapse and develop castration-resistant prostate cancer (CRPC), which still relies on the AR pathway for growth. Tumors at this advanced stage are highly proliferative and metastatic in nature, inevitably leading to fatality due to lack of effective therapies. Growing evidence has shown that ligand-independent AR function in CRPC is regulated through a complex network of co-regulators [1–5]. Their mis-regulation or aberrant expression can impact tumor formation and progression. Widespread studies of AR regulators in advanced prostate cancer have provided a better understanding of mechanisms underlying castration resistance.

Ubiquitously-expressed, prefoldin-like chaperone (UXT) is widely expressed in most human organs. However, its function in human cancer is largely unexplored. UXT has been reported to form an integral component of the NF-κB enhanceosome, which is essential for the nuclear function of NF-κB transcription factor [6]. UXT also interacts with EVI1 to repress cell transformation [7]. Interestingly, interaction between EVI1 and NF-κB has been reported to be important in genomic stability in leukemia [8]. Recent functional studies characterized UXT’s interaction with nuclear receptors, such as ER and AR [9, 10]. Our previous work identified UXT as a transcription factor that directly binds to the AR N-terminus and modulates AR transcriptional activity in prostate cancer cells [10, 11]. Depletion of UXT activates DNA damage checkpoint genes, promotes proliferation, and induces cell resistance to anti-androgen treatment in prostate cancer cells [11]. Depletion of UXT also decreases the protein expression of unconventional prefoldin RBP5 interactor (URI) in prostate cells; we have shown that UXT and URI affect each other’s stability, and in complex together, UXT/URI represses AR mediated transcription and AR occupancy on AR target genes [12, 13].

Analysis of a small clinical cohort suggested that loss of UXT expression in primary prostate cancer may be associated with tumor recurrence and poor prognosis [11]. Here we analyzed a large clinical cohort of prostate cancer to investigate UXT expression in disease progression. We then used prostate-specific *Uxt*^*KO*^ transgenic mice and prostate cancer cell culture models to investigate UXT function in CaP development and progression.

## RESULTS

### Decreased UXT expression in primary prostate cancer is positively associated with tumor recurrence

**Figure 1:**
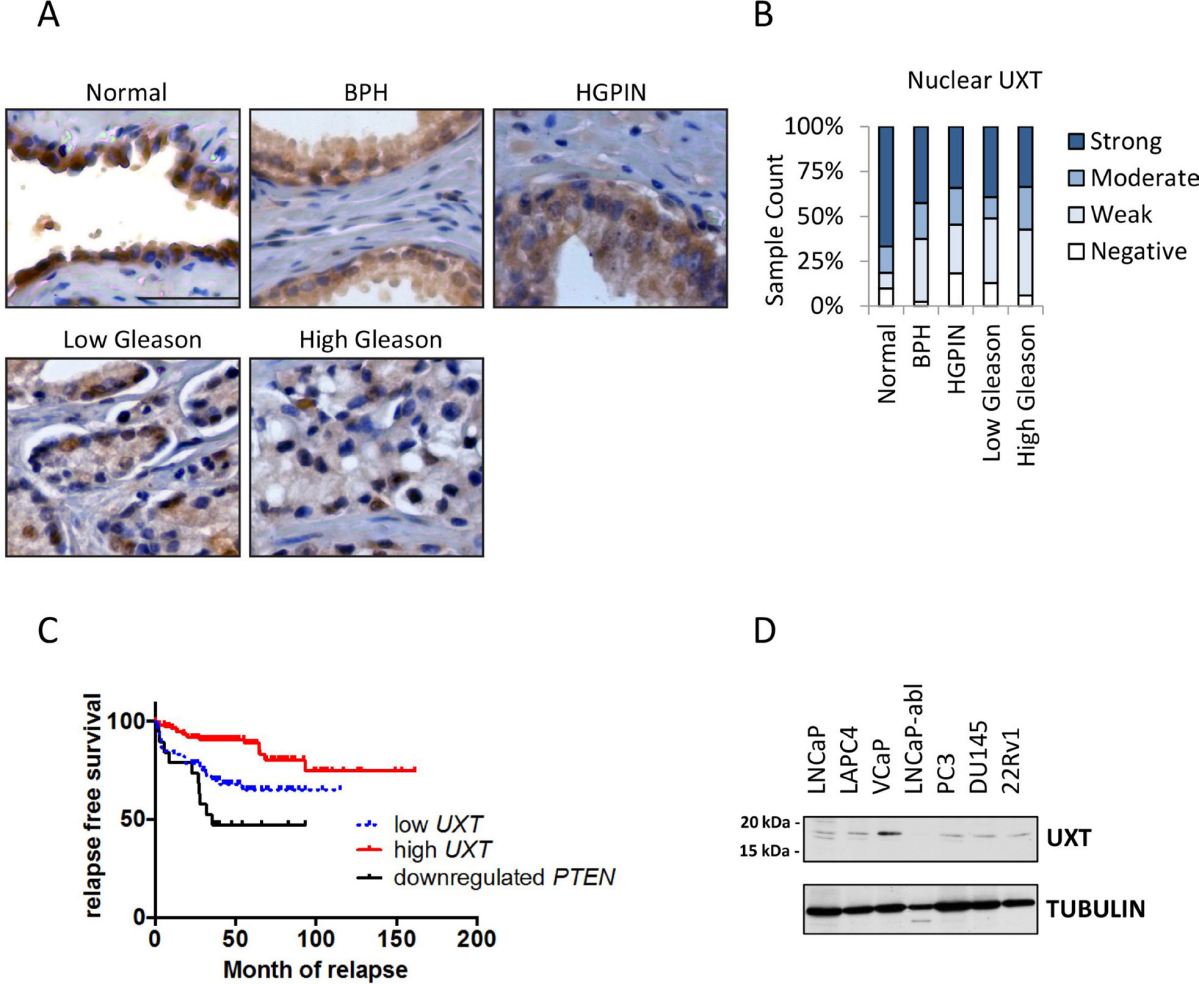
Loss of UXT expression in prostate cancer progression (**A**) IHC analysis in human specimens shows UXT staining in normal prostate gland and prostate lesions with pathological changes. The human samples include normal prostate, BPH, HGPIN, and carcinomas. Scale bar = 10 μm. (**B**) Following IHC analysis, the bar chart quantified the nuclear UXT staining in normal prostate, BPH, HGPIN, Gleason grade 3 cancer, and high Gleason grade (4/5) cancer. (**C**) Kaplan–Meier analysis shows a positive association between *UXT* mRNA expression in primary CaP and patient relapse-free survival. (**D**) Western blots surveyed UXT expression in multiple human prostate cancer cell lines.

To explore the importance of UXT in CaP development, we analyzed UXT protein expression by immunohistochemistry (IHC) in prostate tissues including normal prostate (*n* = 81), benign prostatic hyperplasia (BPH; *n* = 40), high grade prostatic intraepithelial neoplasia (HGPIN; *n* = 44), and 378 primary CaP cases (Supplementary Table 1). IHC showed that, in normal prostate and BPH, UXT is strongly expressed in the epithelial cell nucleus (Figure 1A). While there is no significant difference in UXT expression levels between normal prostate and BPH (*p* = 0.18, Table 1), we found that both HGPIN and malignant tissue have significantly decreased UXT expression compared to normal prostate (*p* = 4.02 × 10^−4^ and *p* = 3 × 10^−6^, respectively) (Table 1 and Figure 1A). Further, UXT protein expression was decreased to a greater extent in tumors with high Gleason scores compared to tumors with low Gleason score in primary CaP (*p* = 0.014; Figure 1A and 1B). Since the degree of UXT loss is associated with increased Gleason score (*p* = 2.65 × 10^−5^), we further examined whether loss of UXT expression in primary tumors is associated with patient prognosis. We retrospectively analyzed *UXT* mRNA levels in 188 primary CaP cases with 12 years of follow up [14]. Patients with low levels of *UXT* have a higher prevalence of developing tumor biochemical recurrence (21/67, 31%) than those with high *UXT* expression levels (15/121, 12.3%, *p* = 1.28 × 10^−10^). Decreased *UXT* levels in primary tumors is also positively associated with poor relapse-free survival (*p* = 0.0017, Figure 1C), where loss of PTEN also has a strong association with poor prognosis (*p* = 3.92 × 10^-4^, Figure 1C). Our data suggest that loss of UXT expression occurs during HGPIN and remains low in cancer. Low levels of UXT in cancer may also play a role in progression as evidenced by biochemical recurrence and poor relapse-free survival. Our previous study has identified UXT as a co-repressor for AR in CaP cells [11]. To explore the impact of loss of UXT in CaP, we first surveyed its protein expression in multiple prostate cancer cell lines. Most cell lines expressed detectable levels of UXT with VCaP cells exhibiting high levels (Figure 1D).

**Table 1:** UXT staining levels in prostate and prostate cancer

	Normal *n* = 81		BPH *n* = 40		HGPIN *n* = 44		Cancer *n* = 378	
Nuclear UXT staining	count	percent	count	percent	count	percent	count	percent
negative	8	9.9%	1	2.5%	8	18.2%	38	10.0%
weak	7	8.6%	14	35%	12	27.3%	138	36.4%
moderate	12	14.8%	8	20%	9	20.5%	63	16.6%
strong	54	66.7%	17	42.5%	15	34.1%	140	36.9%

### *Uxt* knockout in mouse prostate leads to PIN

**Figure 2:**
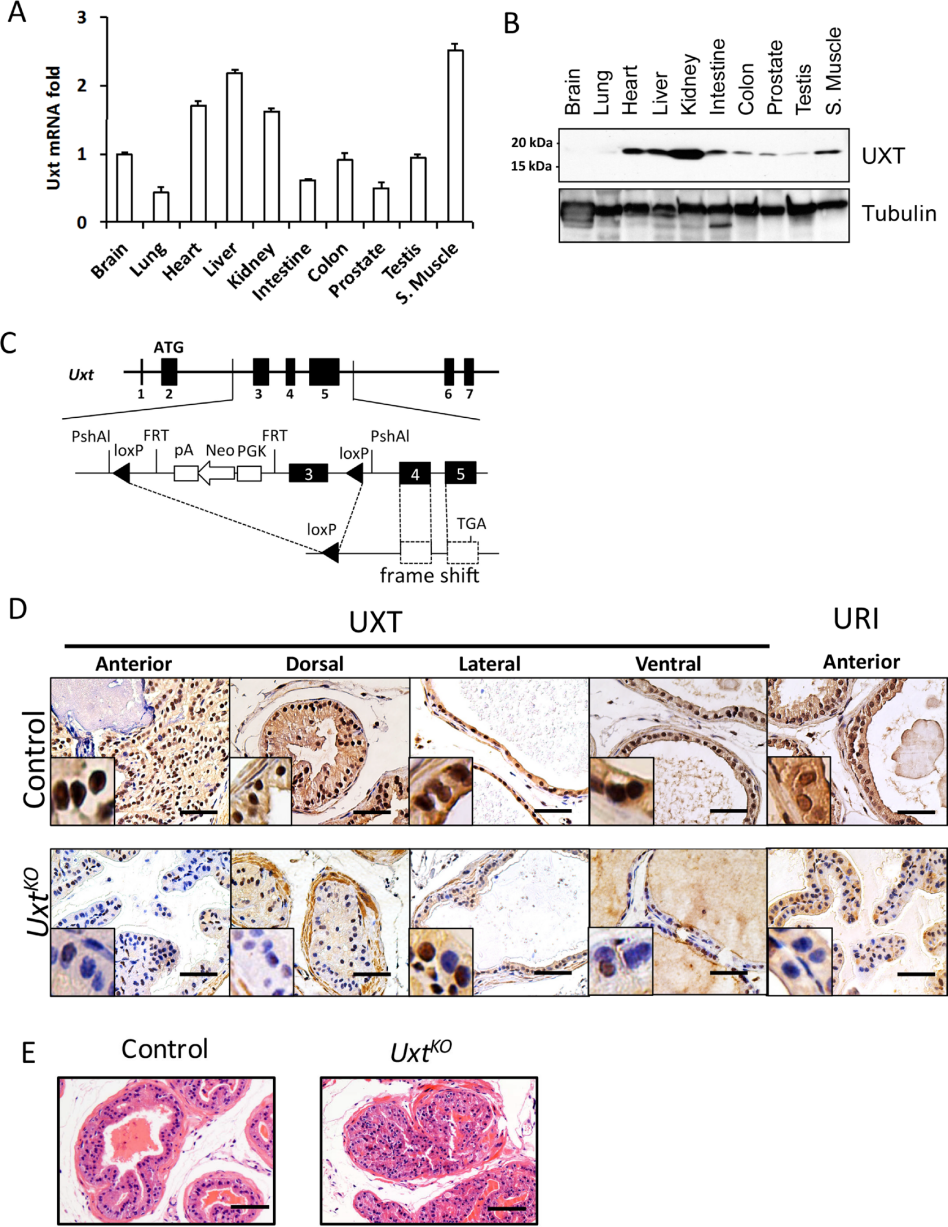
*Uxt* ablation in the mouse prostate resulted in PIN Different mouse organs were harvested for RNA and protein. (**A**) *Uxt* mRNA expression was quantified by qRT-PCR (**B**) UXT protein expression in mouse organs by western blots. Tubulin is included as a loading control. (**C**) Schematic shows the genetic design of Pb-Cre induced *Uxt* ^*KO*^ in mouse prostate. (**D**) IHC with UXT specific antibodies confirmed loss of UXT and URI protein expression in *Uxt*^*KO*^ mouse prostate. (**E**) H&E staining shows a PIN phenotype in *Uxt*^*KO*^ mouse compared to control mice. Scale bar = 100 μm.

We next used an *in vivo* mouse model to determine whether loss of UXT in prostate could promote cell proliferation and accelerate cancer progression. First, we surveyed *Uxt* RNA and UXT protein expression in wild type mice. In mouse, *Uxt* mRNA and UXT protein are ubiquitously expressed in almost all mouse organs including prostate (Figure 2A and 2B). However, expression levels vary among these tissues, with high levels of UXT observed in heart, liver, and kidney (Figure 2B). UXT is expressed in the brain and lung (data not shown) but are below the level of detection using western blot analysis. *Uxt* knockout (KO) in mice is embryonic lethal (data not shown). Therefore, to investigate the impact of UXT loss in CaP initiation/development and progression, we generated a prostate-specific probasin promoter-induced *Uxt Pb-Cre* KO. This *Uxt* mutation, located on the X chromosome, resulted in *Uxt* exon 3 deletion and a frame shift in the downstream coding sequence (Figure 2C). IHC staining of UXT showed that the *Uxt*^*KO*^ mouse lost UXT protein expression in prostate gland epithelial cells in all four regions of the prostate, with high penetration in dorsal and anterior regions (Figure 2D). At 4-months, the *Uxt*^*KO*^ mice exhibited a cribiform PIN phenotype (6/8), which was not observed in littermate controls (0/7) (Figure 2E). The severity of this proliferative phenotype increased with age. At 12-months, 100% (6/6) *Uxt*^*KO*^ mice exhibited PIN compared to control mice (1/5, *p* = 0.0014) (Supplementary Table 2). Our findings in this animal model are consistent with clinical data showing that loss of UXT is an early event during cancer initiation (Figure 1). Consistent with experiments previously conducted in cell culture, the *Uxt*^*KO*^ mice also expressed decreased levels of the UXT binding protein, URI, in prostate (Figure 2D, last column).

**Figure 3:**
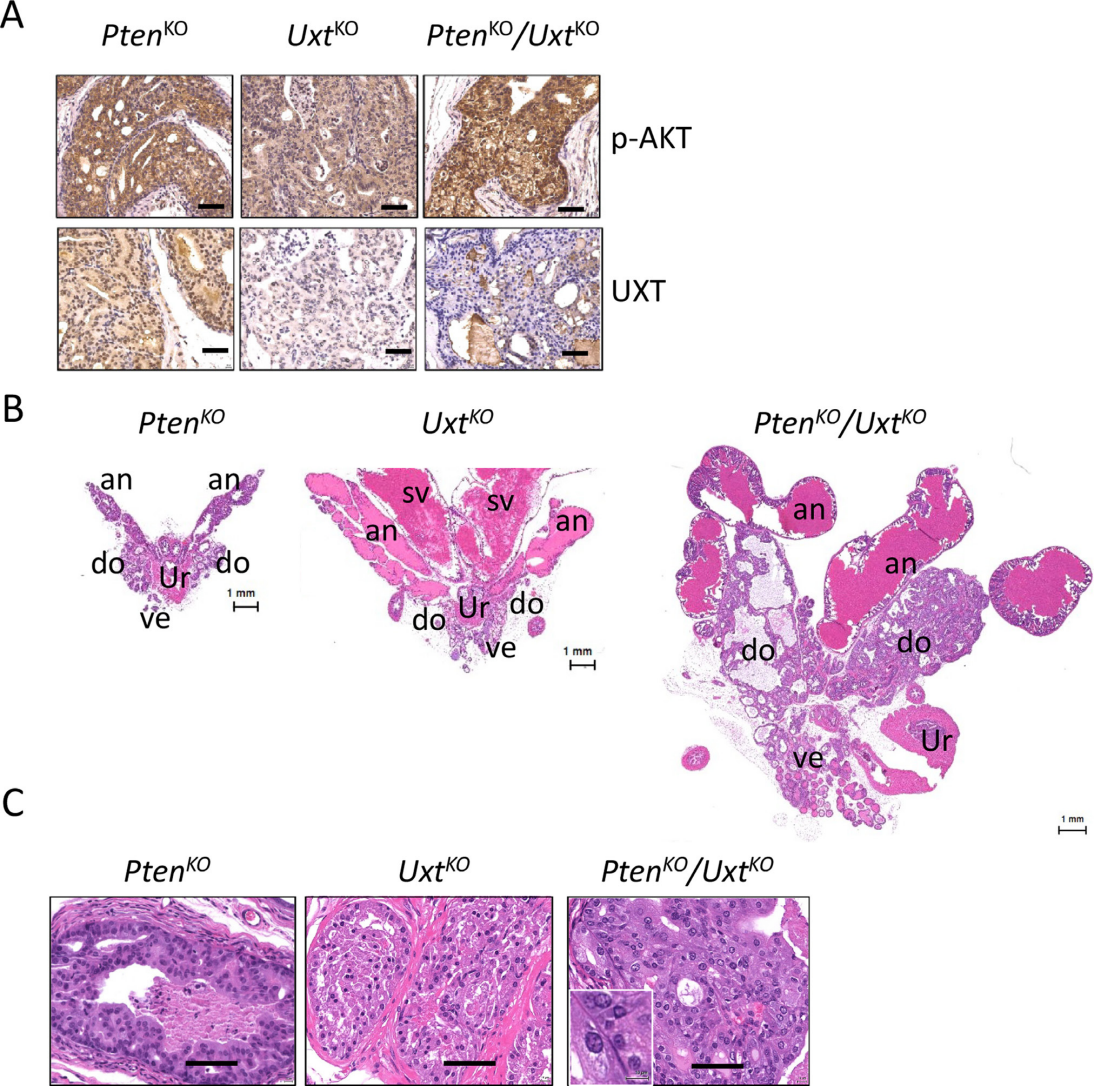
Analysis of *Pten*^*KO*^, *Uxt*^*KO*^, and *Pten/Uxt* double KO mice (**A**) IHC analysis in *Pten*^*KO*^ (*n* = 4), *Uxt*^*KO*^ (*n* = 5), and *Pten*^*KO*^*/Uxt*^*KO*^ (*n* = 3) mice shows phospho-AKT and UXT staining. Scale bar = 100 μm. H&E staining in *Pten*^*KO*^, *Uxt*^*KO*^, and *Pten/Uxt* double KO mice shows enlarged prostates (**B**) and pathological changes (**C**). an: anterior prostate, do: dorsal prostate, ve: ventral prostate, sv: seminal vesicle, ur: urethra. Scale bar = 100 μm. Inset scale bar = 10 μm.

The absence of a malignant phenotype in the *Uxt*^*KO*^ mice suggested that additional genomic alterations are required for prostate cancer development after loss of UXT in prostate. *PTEN* is one of most commonly deleted tumor suppressor genes in HGPIN and CaP [15, 16] and loss of PTEN in CaP is significantly associated with poor prognosis [17]. We reasoned that loss of UXT in the context of PTEN deletion would result in a more severe phenotype than either single deletion. To test this, we generated *Pten/Uxt* double KO mice and compared their phenotype with *Pten*^*KO*^ or *Uxt*^*KO*^ mice (Figure 3A). As expected, the loss of PTEN in both *Pten*^*KO*^ and *Pten*^*KO*^*/Uxt*^*KO*^ resulted in increased AKT phosphorylation compared to that of *Uxt*^*KO*^ mice (Figure 3A).

Grossly, at 6-months, both *Pten*^*KO*^ and *Uxt*^*KO*^ mice exhibited enlarged prostates (Figure 3B). The increased size of the prostate in *Uxt*^*KO*^ mice is due to both tissue enlargement and prostate secretion fluid blockage, resulting in the enlargement of anterior prostate region (Figure 3B, middle). In *Pten*^*KO*^*/Uxt*^*KO*^ mice, both dorsal and ventral prostate are dramatically increased in size compared to that of the single knockout mice (Figure 3B, right). Histological analysis showed papillary hyperplasia (Figure 3C, left) in the *Pten*^*KO*^ mice and cribiform hyperplasia in the *Uxt*^*KO*^ mice (Figure 3C, middle) at 6 months. Distinctively, the *Pten*^*KO*^*/Uxt*^*KO*^ mice have HGPIN indicated by the enlarged epithelial cell nuclei and increased number of nucleoli (Figure 3C, right).

### UXT depletion promotes retrotransposition in prostate cancer

**Figure 4:**
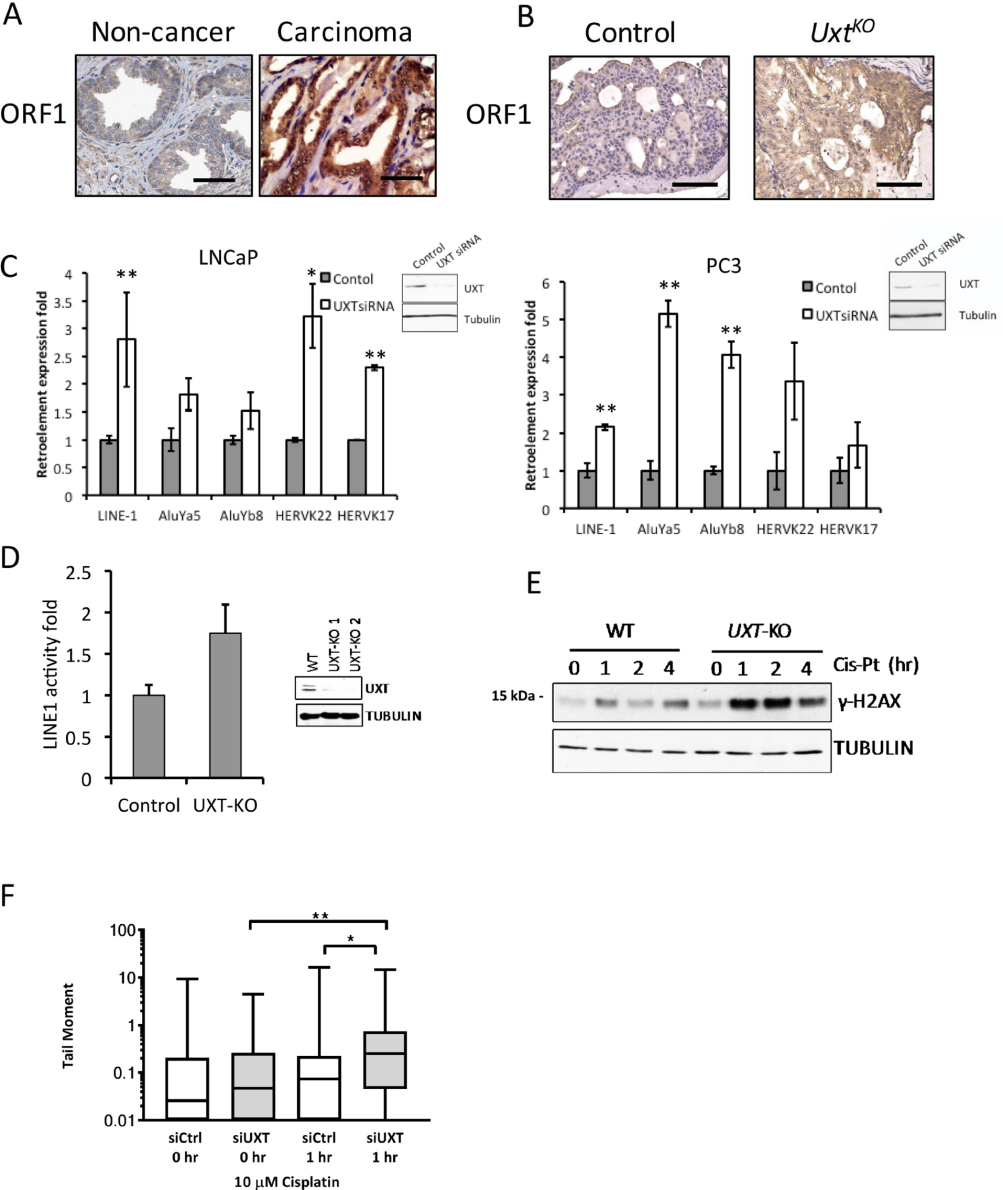
Loss of UXT in prostate promoted LINE-1 retroelement expression and retrotransposition (**A**) IHC analysis shows ORF1 staining in human normal prostate (*n* = 5) and prostate cancer (*n* = 5). (**B**) IHC analysis shows ORF1 expression in control and *Uxt*^*KO*^ mice. Scale bar = 100 µm (**C**) siRNA-mediated UXT knockdown in human prostate cancer cells induced mRNA expression of Alu, HERVK and LINE-1 retroelements. RNA was harvested from control and siUXT LNCaP and PC3 cells. Retroelement expression was quantified by qRT-PCR. Inset panels show loss of UXT protein in UXT-KO cells. ^*^p < 0.05, ^**^p < 0.01 (**D**) Retrotransposition assays show that TALEN-mediated UXT-KO induced retrotransposition in C4-2 cells (*p* = 9 × 10^-4^). Inset shows dramatically decreased UXT protein expression levels. (**E**) UXT-KO in prostate cancer cells increased DNA damage accumulation and sensitized cells to cisplatin. Control and UXT-KO C4-2 cells were treated with cisplatin (10 μM) before cell lysates were harvested for western blot. Unrepaired DNA double strand breaks were measured by phospho-γH2AX by western blots. (**F**) Loss of UXT increases the tail moment in response to cisplatin. C4-2 control (siCtrl) and UXT knockdown (siUXT) cells were treated with cisplatin (10 µM) for 1 hour. The median (line), 25th-75th percentiles (boxes), and minimum and maximum values (whiskers) are shown; ^*^p = 0.0106 and ^**^p = 0.0054.

Genomic instability and increased chromosome rearrangements are very common in advanced prostate cancer especially in metastatic tumors [18, 19]. Although the mechanism of such genome instability is still not fully understood, recent studies have shown that increased retrotransposition activity contributed to the increased genome instability in cancer [20]. Long interspersed element-1 (LINE-1), comprising 17% of the human genomic content [21], is an active mobile genetic element that belongs to the group of non-long terminal repeat (LTR) retrotransposons. LINE-1 is expressed in both germ line and somatic cells, where it contributes to genomic instability via a “copy-and-paste” mechanism of amplification [22]. Full length LINE-1 mRNA contains two open reading frames coding for proteins ORF1p and ORF2p [23]. ORF1 protein (ORF1p) functions as a nucleic acid chaperone that binds LINE-1 mRNA in the cytoplasm during the retrotransposition cycle [24]. ORF2 protein (ORF2p) encodes the endonuclease and reverse transcriptase required for retrotransposition [25, 26]. Our studies showed that the UXT binding partner, URI, transcriptionally represses LINE-1 retroelements by dephosphorylation of the transcriptional repressor KAP1 [27]. Since *Uxt*^*KO*^ animals exhibit decreased URI levels (Figure 2D), we tested whether loss of UXT also resulted in derepression of LINE-1. First, IHC analysis of human prostatectomy samples revealed dramatically increased LINE-1 ORF1 protein levels compared to non-cancerous lesions (Figure 4A). *Uxt*^*KO*^ mice also have increased LINE-1 ORF1 protein levels in prostate compared to controls (Figure 4B). We hypothesized that UXT may repress retroelement expression and that loss of UXT may lead to increased expression and genomic instability. In fact, *UXT* knockdown increased retroelement mRNA expression levels in both AR-positive LNCaP and AR-negative PC3 cells (Figure 4C). The increase in ORF1 expression upon loss of UXT suggests that the cells may be permissive for retrotransposition. To test this, we conducted retrotransposition assays using a Neo-AI-based retrotransposition reporter [28]. These experiments demonstrated that UXT knockdown increased retrotransposition in C4-2 cells (Figure 4D). Further, we observed dramatically increased levels of γ-H2AX (Figure 4E) and increased tail moments (Figure 4F), measured by Comet assays, in prostate cancer cells after UXT depletion and treatment with the DNA intercalating agent cisplatin compared to control cells, indicating that accumulated unpaired DNA double strand breaks are more severe and persist longer upon loss of UXT. The fact that cells with UXT depletion are more sensitive to cisplatin, suggests that UXT may be important to maintain chromosome stability.

## DISCUSSION

Over 40% of the human genome is derived from retroelements. Although the majority of retroelements are inactive due to mutation and truncation, the active LINE-1 retroelements still harbor potential retrotransposition activity that can result in insertional mutations, genome rearrangement, and translocation. Structural alterations in chromosomes are widespread in prostate cancer and contribute to drug resistance as well as poor patient prognosis [14]. In prostate cancer, hypomethylation of retroelements is also associated with cancer progression and is most pronounced in metastatic cases [29, 30]. The mechanism underlying reactivation of normally silenced retroelements in prostate cancer remains unclear. Increased mRNA expression of HERV-K is detected in prostate cancer, and may be involved in chromosomal translocation and cancer initiation [31]. Expression of HERV-K is inducible by androgens in LNCaP cells [32, 33]. This suggests that the AR pathway may increase transcription of retrotransposons. In this study, UXT knockout in prostate cancer cells promoted mRNA expression of both long-interspersed and short-interspersed non-LTR sequences, including LINE-1 and Alu families, respectively (Figure 4C). LINE-1-mediated retrotransposition contributes to genetic plasticity and diversity. Increased ORF1 protein expression levels in *Uxt*^*KO*^ mice prostate tissues supports the idea that a UXT-containing complex may repress retrotransposon expression in the genome. Loss of UXT and concomitant loss of URI expression and its regulation of a repression complex, containing KAP1, HDACs, SETDB1, and HP1, may result in transcriptional de-repression of retroelements in prostate cells [27, 34].

Our previous study in a small cohort linked loss of UXT expression in primary prostate tumors with tumor recurrence [11]. The much larger patient cohort analysis in the current study clearly demonstrates decreased UXT expression during prostate cancer progression. Interestingly, the loss of UXT protein is not limited to advanced prostate cancer, instead, it is commonly found in both BPH and HGPIN. This suggests that UXT expression may act as a protective factor to prevent hyperplasia in prostate. As an early event, loss of UXT may enable unregulated prostate epithelial cell proliferation. This hypothesis is supported by our animal models, in which loss of UXT, similar to *Pten* deletion, alone is sufficient to induce hyperplasia. Interestingly, the prostate hyperplasia in these two types of genetically altered mice has different morphologies with papillary hyperplasia more prevalent in the *Pten* deletion and cribriform hyperplasia more prevalent in the *Uxt* deletion. In addition, the *Uxt* deletion mice exhibited moderate levels of prostate secretion fluid blockage, indicating that the enlargement of prostate resulted in gland obstruction and disfiguration of the organ. Such a BPH-like phenotype was more severe in the *Pten*/*Uxt* double knockout prostate, exhibited by the dramatically increased prostate size. The enlargement of prostate in the *Pten*/*Uxt* mutant mice may have two reasons. First, the hyperplasia is significantly more severe than in single knockout mice. Second, the accumulation of prostate secretion fluid in the anterior prostate and inflation in these glands may be a direct result of the dorsal/ventral obstruction, leading to a further increase in prostate size. In fact, the greatly enlarged prostate in the *Pten*/*Uxt* double knockout occupied the majority of the abdominal cavity and led to infertility (data not shown). Altogether our results suggest that loss of UXT in human prostate may promote genome instability in normal prostate epithelial cells and that loss of UXT as an early event in prostate cancer may lead to chromosomal alterations. The relevance of UXT/PTEN loss in the development of HGPIN and cancer, and determining if loss of UXT is a driver or passenger of eventual cancer development in a subset of HGPIN patients are important questions that still need to be explored. Future studies are also warranted on the genomic instability caused by the loss of UXT, focusing on possible deregulation of UXT interactions with URI, EVI1, and NF-κB, potentially providing cancer cells with a means to evade detection from inflammatory pathways and activation of immune signaling.

## MATERIALS AND METHODS

### Transgenic mouse

*Uxt*^*F/F*^ mice on a 129/SvJ background were generated by Ozgene (Australia). Genomic fragments were amplified by PCR from C57 Bl/6 genomic DNA to generate 5′ and 3′ homology arms, which included exons 1-2 and exons 4–7, respectively. Exon 3 was amplified separately by PCR using primers containing a 3′ loxP site. The exon 3-loxP arm was then cloned downstream of a loxP-FRT-PGK-Neo^R^-FRT selection cassette. Homologous recombination of the targeting vector was carried out in W9.5 ES cells derived from 129/SvJ mice. Clones were selected for neomycin resistance, and DNA from resistant clones was used to test genotyping probes. Correctly targeted W9.5 ES clones were identified by Southern blot analysis and microinjected into C57 Bl/6 blastocysts to generate chimeric animals. After establishment of germline transmission, the FRT-PGK-Neo^R^-FRT cassette was deleted by mating to a transgenic line containing FLP recombinase (Oz-Flp). Oz-Flp was removed by mating *Uxt* ^*floxΔNeo/Y*^; Oz-Flp mice to wild type C57 Bl/6 mice, resulting in *Uxt* ^*F/Y*^ mice of mixed 129/SvJ and C57 Bl/6 background. Mixed background homozygous *Uxt* ^*F/F*^ females were mated to PB-Cre4 males (Jackson Laboratory, Bar Harbor, ME) to generate prostate cell-specific conditional knockouts. *Pten*^*loxp*^ mice were purchased from Jackson Laboratory (Bar Harbor, ME).

### Cell culture and human specimens

Human prostate cancer cell lines were purchased from ATCC (LNCaP, VCaP, PC3, and DU145), and maintained in full media. LNCaP-abl cells were cultured in “androgen-free media” (phenol read-free RPMI1640, 10% charcoal-stripped FBS, 1% Penicillin-Streptomycin). Cells were tested for authentication by STR analysis and also tested for mycoplasma contamination (DDC Medical, Fairfield, OH). Prostate tissue microarray containing normal prostate (*n* = 81), benign prostatic hyperplasia (*n* = 40), HGPIN (*n* = 44), and 378 primary CaP cases from 217 patients was provided by the Prostate Cancer Biorepository Network (PCBN). PCBN also provided detailed patient information including histology, pathology, and prognosis data. UXT protein expression levels in IHC analysis were evaluated blindly by two pathologists, and were categorized as negative, weak, moderate, and strong (Supplementary Figure 1). Patient radical prostatectomy specimens were from NYU School of Medicine. *In silico* analysis of patient prognosis used a cohort of 188 cases previously reported [14]. All patient samples were de-identified. UXT knockdown in human prostate cancer cell lines were achieved by siUXT in transient transfection. The siUXT was from Santa Cruz Biotechnology. Stable depletion of UXT expression in human prostate cancer cells was achieved through XTN TALENs-mediated genomic mutation. The XTN constructs for site-specific mutation in UXT exon 3 was created by Transposagen, LLC (Lexington, KY). This mutation generated an early stop codon and frame shift in the *UXT* gene on the X chromosome.

### Western blotting

Western blotting was performed as described elsewhere [35]. Rabbit polyclonal anti-human UXT, anti-mouse UXT, and anti-URI antibodies were described previously [12]. Rabbit polyclonal anti-phospho-AKT antibody was from Cell Signaling. Rabbit anti-mouse ORF1 antibody was kindly provided by Emily Adney and Dr. Jef Boeke (NYU School of Medicine). Mouse monoclonal anti-alpha-tubulin antibody was purchased from Sigma-Aldrich, St. Louis, MO.

### Retrotransposition assay

Measurement of retrotransposition activity with Neo-AI indicators followed the protocol described previously [36]. C4-2 cells were seeded at 3 × 10^6^ per well in a 100 mm plate, transfected with FuGene HD (Roche Applied Science, Indianapolis, IN USA) on the following day, and selected by growing with puromycin 2.5 μg/ml for 3 days. For each transfection, three 100 mm dishes were seeded with 1×10^5^ cells each under G418 selection (500 μg/ml) for 10 to 14 days. Cells were washed in 1x phosphate-buffered saline (PBS) and were fixed to plates by treatment with FIX solution (acetic acid: methanol 1:2) for 30 min. We stained the fixed cells with 0.5% crystal violet at room temperature for 10 min, washed them with PBS. Surviving cell clones were then counted.

### Comet assay

C4-2 cells in the presence or absence of UXT were treated with 10 µM cisplatin for one hour and then subjected to single cell gel electrophoresis under alkaline conditions according the manufacturer’s instructions (Trevigen, Gaithersburg, MD). Greater than 50 cells were counted per condition and tail moments (tail length × percentage of DNA in tail) quantified using ImageJ v1.49 (NIH, Bethesda, MD) and Open Comet v1.3 [37].

### Statistical analysis

Relapse free survival was calculated using the Kaplan–Meier method, and the significance between groups was calculated by Wilcoxon test. Expression of UXT levels in different human prostate tissues and Comet assays was analyzed with one-way ANOVA. The retroelement expression and LINE-1 activity were analyzed by *t*-test. Statistical analyses were performed using Prism version 6.0 (GraphPad Software, La Jolla, CA), and SPSS version 13.0 (SPSS, Inc., Chicago, IL). Data cleaning were performed by cBioPortal [38]. All *in vitro* experiments were performed three times independently and the error bars represent the standard deviation with significance calculated by nonparametric *t*-test.

## SUPPLEMENTARY MATERIALS FIGURE AND TABLES



## References

[1] Lee E, Madar A, David G, Garabedian MJ, Dasgupta R, Logan SK (2013). Inhibition of androgen receptor and beta-catenin activity in prostate cancer. Proc Natl Acad Sci U S A.

[2] Wang Y, Kreisberg JI, Ghosh PM (2007). Cross-talk between the androgen receptor and the phosphatidylinositol 3-kinase/Akt pathway in prostate cancer. Curr Cancer Drug Targets.

[3] Gelman IH, Peresie J, Eng KH, Foster BA (2014). Differential requirement for Src family tyrosine kinases in the initiation, progression, and metastasis of prostate cancer. Mol Cancer Res.

[4] Augello MA, Hickey TE, Knudsen KE (2011). FOXA1: master of steroid receptor function in cancer. EMBO J.

[5] Miyamoto DT, Zheng Y, Wittner BS, Lee RJ, Zhu H, Broderick KT, Desai R, Fox DB, Brannigan BW, Trautwein J, Arora KS, Desai N, Dahl DM (2015). RNA-Seq of single prostate CTCs implicates noncanonical Wnt signaling in antiandrogen resistance. Science.

[6] Sun S, Tang Y, Lou X, Zhu L, Yang K, Zhang B, Shi H, Wang C (2007). UXT is a novel and essential cofactor in the NF-kappaB transcriptional enhanceosome. J Cell Biol.

[7] McGilvray R, Walker M, Bartholomew C (2007). UXT interacts with the transcriptional repressor protein EVI1 and suppresses cell transformation. FEBS J.

[8] Colombo AR, Zubair A, Thiagarajan D, Nuzhdin S, Triche TJ, Ramsingh G (2017). Suppression of Transposable Elements in Leukemic Stem Cells. Sci Rep.

[9] Sanchez-Morgan N, Kirsch KH, Trackman PC, Sonenshein GE (2017). UXT Is a LOX-PP Interacting Protein That Modulates Estrogen Receptor Alpha Activity in Breast Cancer Cells. J Cell Biochem.

[10] Markus SM, Taneja SS, Logan SK, Li W, Ha S, Hittelman AB, Rogatsky I, Garabedian MJ (2002). Identification and characterization of ART-27, a novel coactivator for the androgen receptor N terminus. Mol Biol Cell.

[11] Nwachukwu JC, Mita P, Ruoff R, Ha S, Wang Q, Huang SJ, Taneja SS, Brown M, Gerald WL, Garabedian MJ, Logan SK (2009). Genome-wide impact of androgen receptor trapped clone-27 loss on androgen-regulated transcription in prostate cancer cells. Cancer Res.

[12] Mita P, Savas JN, Djouder N, Yates JR, Ha S, Ruoff R, Schafler ED, Nwachukwu JC, Tanese N, Cowan NJ, Zavadil J, Garabedian MJ, Logan SK (2011). Regulation of androgen receptor-mediated transcription by RPB5 binding protein URI/RMP. Mol Cell Biol.

[13] Mita P, Savas JN, Ha S, Djouder N, Yates JR, Logan SK (2013). Analysis of URI nuclear interaction with RPB5 and components of the R2TP/prefoldin-like complex. PLoS One.

[14] Taylor BS, Schultz N, Hieronymus H, Gopalan A, Xiao Y, Carver BS, Arora VK, Kaushik P, Cerami E, Reva B, Antipin Y, Mitsiades N, Landers T (2010). Integrative genomic profiling of human prostate cancer. Cancer Cell.

[15] Feilotter HE, Nagai MA, Boag AH, Eng C, Mulligan LM (1998). Analysis of PTEN and the 10q23 region in primary prostate carcinomas. Oncogene.

[16] Verhagen PC, van Duijn PW, Hermans KG, Looijenga LH, van Gurp RJ, Stoop H, van der Kwast TH, Trapman J (2006). The PTEN gene in locally progressive prostate cancer is preferentially inactivated by bi-allelic gene deletion. J Pathol.

[17] Koksal IT, Dirice E, Yasar D, Sanlioglu AD, Ciftcioglu A, Gulkesen KH, Ozes NO, Baykara M, Luleci G, Sanlioglu S (2004). The assessment of PTEN tumor suppressor gene in combination with Gleason scoring and serum PSA to evaluate progression of prostate carcinoma. Urol Oncol.

[18] Barbieri CE, Demichelis F, Rubin MA (2012). Molecular genetics of prostate cancer: emerging appreciation of genetic complexity. Histopathology.

[19] Beltran H, Yelensky R, Frampton GM, Park K, Downing SR, MacDonald TY, Jarosz M, Lipson D, Tagawa ST, Nanus DM, Stephens PJ, Mosquera JM, Cronin MT (2013). Targeted next-generation sequencing of advanced prostate cancer identifies potential therapeutic targets and disease heterogeneity. Eur Urol.

[20] Rodic N, Burns KH (2013). Long interspersed element-1 (LINE-1): passenger or driver in human neoplasms?. PLoS Genet.

[21] Lander ES, Linton LM, Birren B, Nusbaum C, Zody MC, Baldwin J, Devon K, Dewar K, Doyle M, FitzHugh W, Funke R, Gage D, Harris K (2001). Initial sequencing and analysis of the human genome. Nature.

[22] Bibillo A, Eickbush TH (2002). The reverse transcriptase of the R2 non-LTR retrotransposon: continuous synthesis of cDNA on non-continuous RNA templates. J Mol Biol.

[23] Scott AF, Schmeckpeper BJ, Abdelrazik M, Comey CT, O’Hara B, Rossiter JP, Cooley T, Heath P, Smith KD, Margolet L (1987). Origin of the human L1 elements: proposed progenitor genes deduced from a consensus DNA sequence. Genomics.

[24] Khazina E, Truffault V, Buttner R, Schmidt S, Coles M, Weichenrieder O (2011). Trimeric structure and flexibility of the L1ORF1 protein in human L1 retrotransposition. Nat Struct Mol Biol.

[25] Feng Q, Moran JV, Kazazian HH, Boeke JD (1996). Human L1 retrotransposon encodes a conserved endonuclease required for retrotransposition. Cell.

[26] Mathias SL, Scott AF, Kazazian HH, Boeke JD, Gabriel A (1991). Reverse transcriptase encoded by a human transposable element. Science.

[27] Mita P, Savas JN, Briggs EM, Ha S, Gnanakkan V, Yates JR, Robins DM, David G, Boeke JD, Garabedian MJ, Logan SK (2016). URI Regulates KAP1 Phosphorylation and Transcriptional Repression via PP2A Phosphatase in Prostate Cancer Cells. J Biol Chem.

[28] Ostertag EM, Prak ET, DeBerardinis RJ, Moran JV, Kazazian HH (2000). Determination of L1 retrotransposition kinetics in cultured cells. Nucleic Acids Res.

[29] Yegnasubramanian S, Haffner MC, Zhang Y, Gurel B, Cornish TC, Wu Z, Irizarry RA, Morgan J, Hicks J, DeWeese TL, Isaacs WB, Bova GS, De Marzo AM (2008). DNA hypomethylation arises later in prostate cancer progression than CpG island hypermethylation and contributes to metastatic tumor heterogeneity. Cancer Res.

[30] Florl AR, Steinhoff C, Muller M, Seifert HH, Hader C, Engers R, Ackermann R, Schulz WA (2004). Coordinate hypermethylation at specific genes in prostate carcinoma precedes LINE-1 hypomethylation. Br J Cancer.

[31] Perot P, Cheynet V, Decaussin-Petrucci M, Oriol G, Mugnier N, Rodriguez-Lafrasse C, Ruffion A, Mallet F (2013). Microarray-based identification of individual HERV loci expression: application to biomarker discovery in prostate cancer. J Vis Exp.

[32] Hermans KG, van der Korput HA, van Marion R, van de Wijngaart DJ, Ziel-van der Made A, Dits NF, Boormans JL, van der Kwast TH, van Dekken H, Bangma CH, Korsten H, Kraaij R, Jenster G (2008). Truncated ETV1, fused to novel tissue-specific genes, and full-length ETV1 in prostate cancer. Cancer Res.

[33] Tomlins SA, Laxman B, Dhanasekaran SM, Helgeson BE, Cao X, Morris DS, Menon A, Jing X, Cao Q, Han B, Yu J, Wang L, Montie JE (2007). Distinct classes of chromosomal rearrangements create oncogenic ETS gene fusions in prostate cancer. Nature.

[34] Friedman JR, Fredericks WJ, Jensen DE, Speicher DW, Huang XP, Neilson EG, Rauscher FJ (1996). KAP-1, a novel corepressor for the highly conserved KRAB repression domain. Genes Dev.

[35] Wang Y, Mikhailova M, Bose S, Pan CX, deVere White RW, Ghosh PM (2008). Regulation of androgen receptor transcriptional activity by rapamycin in prostate cancer cell proliferation and survival. Oncogene.

[36] An W, Dai L, Niewiadomska AM, Yetil A, O’Donnell KA, Han JS, Boeke JD (2011). Characterization of a synthetic human LINE-1 retrotransposon ORFeus-Hs. Mob DNA.

[37] Gyori BM, Venkatachalam G, Thiagarajan PS, Hsu D, Clement MV (2014). OpenComet: an automated tool for comet assay image analysis. Redox Biol.

[38] Cerami E, Gao J, Dogrusoz U, Gross BE, Sumer SO, Aksoy BA, Jacobsen A, Byrne CJ, Heuer ML, Larsson E, Antipin Y, Reva B, Goldberg AP (2012). The cBio cancer genomics portal: an open platform for exploring multidimensional cancer genomics data. Cancer Discov.

