# Supplementary material for: Prostate-specific loss of UXT promotes cancer progression
Source: Oncotarget. 2019 Jan 22;10(7):707–16. doi: 10.18632/oncotarget.26573 (PMC6366831; doi:10.18632/oncotarget.26573)
Supplement: Supplementary file 1 [file oncotarget-10-707-s001.pdf]

Prostate-specific loss of UXT promotes cancer progression

SUPPLEMENTARY MATERIALS

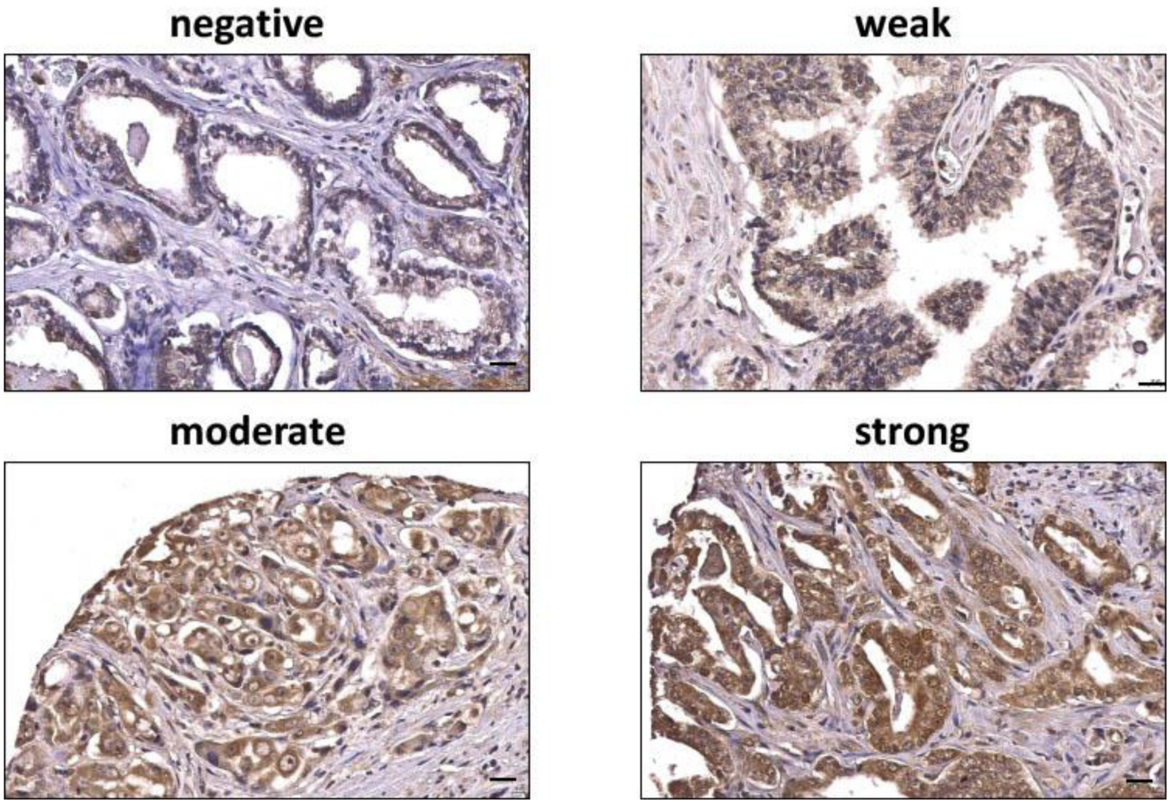

Supplementary Figure 1: UXT expression levels in human prostate. Human prostate specimens from PCBN were subjected to IHC analysis with rabbit anti-human UXT antibody. UXT expression level was categorized as negative, weak, moderate, or strong.

Supplementary Table 1: Clinicopathologic characteristics of overall cohort

**Age (y)**	**Mean (Std. Dev.)**	**60.5 ± 7.1**
PSA at diagnosis (ng/mL)	Mean (Std. Dev.)	6.8 ± 6.0
Clinical T Stage	T1	127 (62.3%)
	T2	33 (16.2%)
	Tx/Unknown	44 (21.5%)
Pathological T Stage	pT2	138 (67.6%)
	pT3	65 (31.9%)
Gleason Sum	5	2 (1.0%)
	6	68 (33.3%)
	7	108 (52.8%)
	8	8 (3.9%)
	9	18 (8.8%)
Surgical Margins	Negative	171 (83.8%)
	Positive	33 (16.2%)
Extension	Negative	198 (97.1%)
	Positive	6 (2.9%)
SVI	Negative	186 (91.2%)
	Positive	18 (8.8%)
Outcome (%)	Nil Relapse	164 (80.4%)
	Relapse	38 (18.6%)
	Lost Follow-up	2 (1.0%)
Length of Follow-up	Mean (months)	82
Period Disease Free	Mean (months)	31
Race	African-American	5
	Asian	5
	Other	7
	Caucasian	176
	Unknown	8

Supplementary Table 2: Mouse prostate pathological changes

**Age (months)**		***Uxt$^{Flox/Y}$***	***Uxt$^{Flox/Y}$, Cre***
4-6		0/7	6/8
12		1/5	6/6
	Total	1/12	12/14
